# Increasing AR by HIF-2*α* inhibitor (PT-2385) overcomes the side-effects of sorafenib by suppressing hepatocellular carcinoma invasion via alteration of pSTAT3, pAKT and pERK signals

**DOI:** 10.1038/cddis.2017.411

**Published:** 2017-10-12

**Authors:** Junjie Xu, Longbo Zheng, Jiang Chen, Yin Sun, Hui Lin, Ren-an Jin, Minyue Tang, Xiao Liang, Xiujun Cai

**Affiliations:** 1 Key Laboratory of Endoscopic Technique Research of Zhejiang Province, Department of General Surgery, Sir Run-Run Shaw Hospital, Zhejiang University, Hangzhou 310016, China; 2 Department of Radiation Oncology, University of Rochester Medical Center, Rochester, NY 14642, USA; 3 Department of Reproductive Endocrinology, Women's Hospital, School of Medicine, Zhejiang University, Hangzhou 310006, China

## Abstract

Although sorafenib is currently used as a standard treatment for advanced hepatocellular carcinoma, low response rate, transient and limited efficacy, primary and acquired resistance and negative side-effects gain increasing attentions, suggesting the need for better efficacious combination therapy. Here, we demonstrated that the sorafenib-induced or hypoxia-induced hypoxia inducible factor (HIF)-2*α* could bind to an hypoxia responsive element within 500 bp region of androgen receptor (AR) promoter and thus transcriptionally suppress AR. Importantly, *In vitro* and *In vivo* studies suggested a specific and potent HIF-2*α* inhibitor, PT-2385, could significantly enhance sorafenib efficacy by suppressing HIF-2*α*, increasing AR and suppressing downstream pSTAT3/pAKT/pERK pathways. Clinical samples further confirmed the role of HIF-2*α* and AR. It is promising that PT-2385 could alleviate the undesirable side-effects of sorafenib treatment by sorafenib-PT-2385 combination therapy, which may shed light for late-stage HCC patients.

Hepatocellular carcinoma (HCC), the most common primary liver tumor with globally increasing incidence, is listed as the sixth most frequently diagnosed cancer and the third most common cause of cancer-related death.^1, 2^ Although targeted chemotherapy developed quite fast in recent years, for late-stage HCC patients who become unqualified for liver transplantation or resection, the management is still quite a challenge.

As the first FDA-approved targeted drug for advanced HCC, sorafenib is a tyrosine kinase inhibitor that targets VEGFR2 and Raf kinase.^3^ Although Phase III clinical trials revealed considerable survival benefit with sorafenib treatment,^4, 5^ there were still numerous HCC patients who poorly responded or even developed resistance after several months treatment.^6^ Recently, increasing studies suggested primary and acquired resistance of sorafenib with activated downstream signals.^7, 8, 9, 10^ Indeed, previous studies found enhanced tumor progression and increased metastasis after sorafenib initial antitumor activity in mouse models.^11, 12^ The transient and limited efficacy may be due, at least in part, to the induced tumor hypoxia and activated compensatory survival signals by sustained sorafenib anti-angiogenic treatment.^13^ Thus, the sorafenib combination therapy with a ‘sensitizer’ that can suppress hypoxia-mediated effects is in urgent need.

The androgen receptor (AR), a ligand-dependent transcriptional factor, plays important roles in the development, progression and metastasis of HCC.^14^ With a male predominance in HCC, previous studies suggested that androgen/AR signals might promote hepatocarcinogenesis.^15^ However, recent studies using liver-specific deletion of AR mice model indicated the dual roles of AR showing AR could promote HCC initiation and development at early stages, yet suppress metastasis in the advanced stages of HCC.^16^ Potential molecules or drugs that could increase AR expression to enhance sorafenib efficacy were recently studied,^17^ but still remained to be further explored.

In the present study, we found sorafenib could induce hypoxia inducible factor (HIF)-2*α*, which transcriptionally decreased AR expression, whereas HIF-2*α* Inhibitor, PT-2385, could overcome the side-effects of sorafenib by suppressing HIF-2*α* and upregulating AR to enhance sorafenib efficacy on HCC invasion via alteration of pSTAT3, pAKT and pERK Signals *in vitro* and *in vivo*.

## Results

### AR enhances sorafenib efficacy to suppress HCC invasion *via* AR-pSTAT3/pAKT/pERK pathway

**Figure 1:**
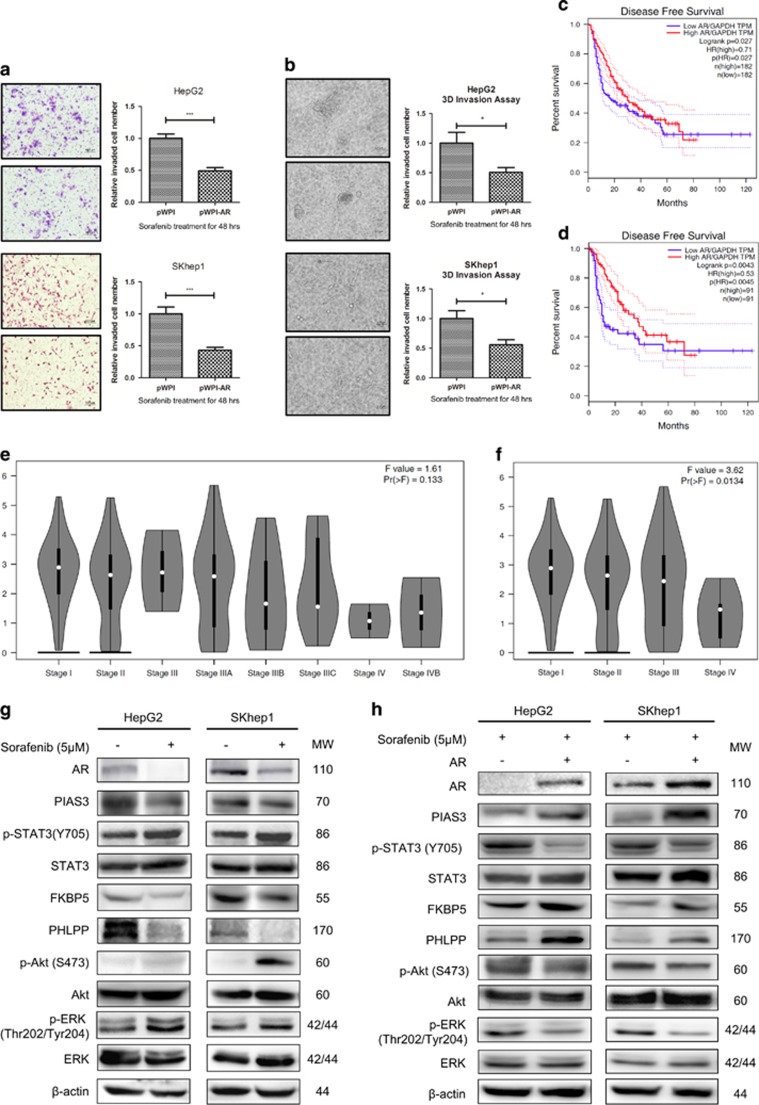
AR enhances sorafenib efficacy to suppress HCC invasion *via* AR-pSTAT3/pAKT/pERK pathway. (**a**) Chamber-transwell invasion assays showed that overexpression of AR decreased the cell invasion in HepG2 and SKhep1 cells under 48 h sorafenib (5 *μ*M) treatment. Left panel, representative images of the chamber-transwell invasion assays; right panel, quantification of the invaded cells. The invaded cells were counted in 10 randomly chosen microscopic fields (× 100) of each experiment and pooled. (**b**) 3D invasion assays on HepG2 and SKhep1 cells showed that overexpression of AR could significantly decrease the invasion ability under 48 h sorafenib (5 *μ*M) treatment. The cells with protrusions were regarded as invaded cells and 10 random different fields at × 200 magnification were counted for quantification. (**c**) Disease-free survival (DFS) curve of HCC patients (*N*=364) from TCGA project indicated that patients with higher AR expression (defined by RNA sequencing with group cutoff in median) had significant better disease-free survival (HR=0.71) than patients lower AR expression. (**d**) Disease-free survival (DFS) curve of HCC patients (*N*=182) indicated that patients with higher AR expression (defined by RNA sequencing with group cutoff in 75%/25% quartile) had significant better disease-free survival (HR=0.53) than patients lower AR expression. (**e**) Sub-stage-dependent analysis suggested lower AR expression in later stages of HCC patients from TCGA project. (**f**) Major-stage-dependent analysis lower AR expression in later stages of HCC patients from TCGA project. (**g**) Western blot assays were used to test downstream altered molecules upon 5 *μ*M treatment in SKhep1 and HA22T cells. (**h**) Western blot assays were used to test downstream altered molecules upon overexpressing AR in SKhep1 and HA22T cells under 48 h sorafenib treatment. *P*<0.05 was considered statistically significant. * *P*<0.05 and *** *P*<0.001

Early *in vitro* cell line studies and *in vivo* mice studies suggested that increased AR expression with a moderate dose (5 *μ*M) of sorafenib might result in higher efficacy of HCC suppression at late stages.^16^ To further verify the effect of AR on the invasion of HCC cells, chamber-transwell invasion assays were performed and results showed that, in the presence of moderate dose of sorafenib (5 *μ*M), overexpression of AR decreased the cell invasion in HepG2, SKhep1 and Huh7 (Figure 1a and Supplementary Figure S1A), consistent with the previous study.^16^ Moreover, 3D invasion assays showed the similar results (Figure 1b), suggesting that AR could function as a late-stage HCC metastasis suppressor under sorafenib treatment. Moreover, analysis of the RNA sequencing expression data of the HCC patients from the TCGA project^18^ indicated that the patients with lower AR expression had worse disease-free survival (DFS), compared with those with higher AR expression (group cutoff at median in Figure 1c and group cutoff at 75%/25% in Figure). Importantly, the stage-dependent analysis also suggested that AR was lower expressed in later stages, indicating the metastasis suppressor role of AR in advanced-stage HCC patients (sub-stage analysis in Figure 1e and major-stage analysis in Figure 1f).

Previous study indicated that pSTAT3 (Y705) bounced under the treatment of sorafenib (5 *μ*M),^19^ and many recent studies focused on the role of pSTAT3 (Y705) in the development of sorafenib resistance in HCC cell lines.^20, 21, 22^ However, the latent detailed mechanism remained to be elucidated. In this study, we found AR significantly decreased when treated with sorafenib (5 *μ*M) in HepG2 and SKhep1 cells (Figure 1g). As indicated by early studies, androgen/AR signaling could induce the expression of protein inhibitor of activated STAT3 (PIAS3), which could suppress the STAT3 activation in prostate cancer.^23^ Thus, we checked the expression level of PIAS3 and it was found that PIAS3 was decreased by sorafenib treatment, which possibly contributed to the increase of pSTAT3 (Y705) (Figure 1g). Moreover, previous study demonstrated that HCC cell invasion could be suppressed via altering the AR-FKBP5-PHLPP-(pAKT and pERK) signals.^17^ Here in the present study, FKBP5 and PHLPP were decreased and pAKT (S473) and pERK (Thr202/Tyr204) were hence increased by sorafenib treatment.

To see if AR indeed has a role in the hypothesized pathway, an interruption approach via overexpressing AR revealed that the increase of AR reversed the expression of PIAS3, pSTAT3 (Y705), FKBP5, PHLPP, pAKT (S473) and pERK (Thr202/Tyr204) in HepG2, SKhep1 and Huh7 cells (Figure 1h and Supplementary Figure S1B), indicating that AR could enhance sorafenib efficacy to suppress HCC invasion via AR-pSTAT3/pAKT/pERK pathway.

### Sorafenib decreases AR by upregulating HIF-2*α*, which transcriptionally regulates AR

**Figure 2:**
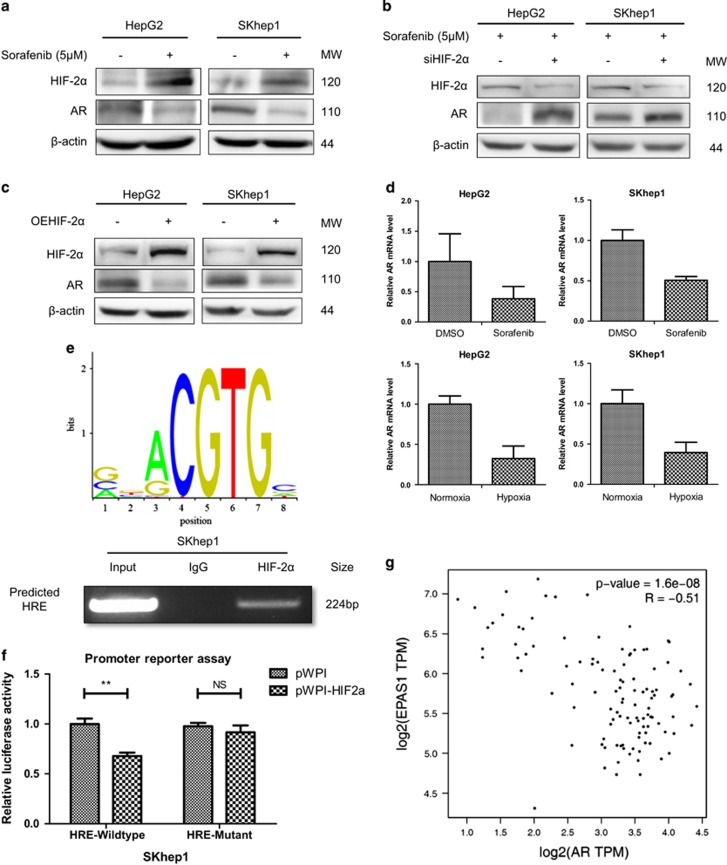
Sorafenib decreases AR by upregulating HIF-2*α*, which transcriptionally regulates AR. (**a**) HIF-2*α* and AR protein levels were checked by western blot assays, showing sorafenib (5 *μ*M) treatment for 48 h could increase HIF-2*α* and decrease AR expression. (**b**, **c**) Western blot assays found that knocking down of HIF-2*α* could increase AR protein level under 48 h sorafenib (5 *μ*M) treatment, whereas overexpression of HIF-2*α* could decrease AR protein level. (**d**) AR mRNA levels were checked by qRT-PCR assays showing that AR mRNA levels could be significantly decreased by sorafenib treatment or hypoxia condition. (**e**) Upper panel: HRE motif sequences from JASPER Database; lower panel: chromatin immunoprecipitation (ChIP) assay showed HIF-2*α* could physically bind to the predicted HRE sequence. (**f**) Promoter reporter assay and mutation rescue assay suggested that HIF-2*α* could inhibit AR promoter activity by specifically interacting with the predicted HRE. (**g**) Correlation analysis of the RNA sequencing expression data from the GTEx project showed that AR negatively correlate with HIF-2*α* (also named as EPAS1) in HCC patients. *P*<0.05 was considered statistically significant. ** *P*<0.01

Although AR was reported to be downregulated by sorafenib treatment in HCC^24^ and prostate cancer,^25^ the underlying mechanism how sorafenib decreases AR still remained elusive. With anti-angiogenic effects, sorafenib was reported to cause hypoxia and therefore induce resistance as a cytoprotective adaptive response.^13, 26^ In this study, we found HIF-2*α* was significantly increased when treated with sorafenib (5 *μ*M) (Figure 2a and Supplementary Figure S1D), and it was possible that the induced HIF-2*α* contributed to the AR reduction. To clarify the impact of HIF-2*α* on AR, we manipulated HIF-2*α* expression in HCC cells. It was found that knocking down of HIF-2*α* could increase AR protein level under sorafenib (5 *μ*M) treatment (Figure 2b and Supplementary Figure S1C), whereas overexpression of HIF-2*α* could decrease AR protein level (Figure 2c). To further verify the mechanism of HIF-2*α*-AR regulation, we checked the AR mRNA level when treated with sorafenib or under hypoxia condition and found AR mRNA levels were significantly decreased under sorafenib treatment or hypoxia condition (Figure 2d), suggesting AR might be transcriptional regulated by HIF-2*α*. Moreover, we found there was a hypoxia responsive element (HRE) within 500 bp region of AR promoter, therefore hypothesized that HIF-2*α* could bind to this HRE and transcriptional inhibit the AR expression. Chromatin immunoprecipitation (ChIP) assay showed HIF-2*α* could physically bind to the predicted HRE sequence (Figure 2e), and promoter reporter assay and mutation rescue assay further suggested HIF-2*α* could inhibit AR promoter activity by specifically interacting with the predicted HRE (Figure 2f, detailed sequence information see Supplementary Table 1). Importantly, the correlation analysis of the RNA sequencing expression data from the GTEx project^18^ showed that AR negatively correlate with HIF-2*α* (also named as EPAS1) in HCC patients (Figure 2g).

### Hypoxia decreases AR by upregulating HIF-2*α*

**Figure 3:**
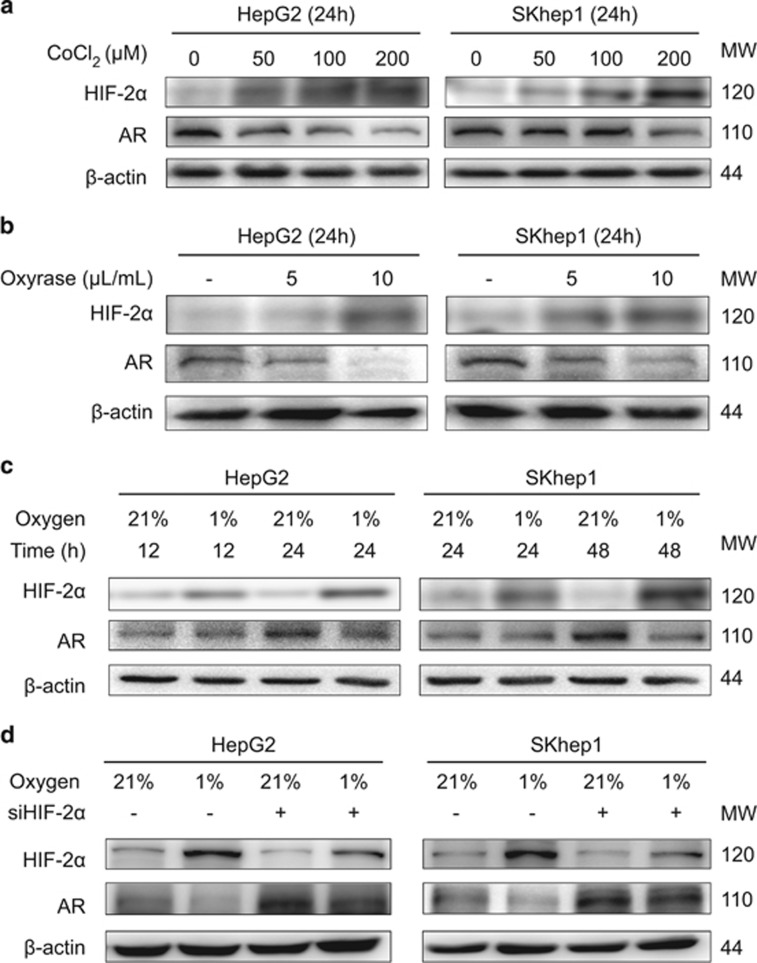
Hypoxia decreases AR by upregulating HIF-2*α*. (**a**,**b**) Western blot assays showed that cobalt chloride and Oxyrase treatments could suppress AR expression on HepG2 and SKhep1 as the treatment being prolonged. (**c**) Western blot assays showed exposure to hypoxia (1% oxygen) could significantly suppress AR expression in both HepG2 and SKhep1 cells. (**d**) Western blot assays showed knocking down of HIF-2*α* could partially rescued the AR decreased by hypoxia

As suggested by previous results showing HIF-2*α* could transcriptionally inhibit AR, it was possible that hypoxia condition could decrease AR level by upregulating HIF-2*α*. Cobalt chloride and oxyrase treatments mimicking hypoxia conditions on HepG2 and SKhep1 cells showed significant AR suppression as the treatment being prolonged (Figures 3a and b). Moreover, exposure to hypoxia (1% oxygen) also significantly suppressed AR expression in both HepG2 and SKhep1 cells (Figure 3c), which could be partially rescued by knocking down of HIF-2*α* (Figure 3d), indicating that hypoxia could decrease AR by upregulating HIF-2*α*.

### PT-2385 induces AR by suppressing HIF-2*α* and enhances sorafenib efficacy to suppress HCC invasion *in vitro* and *in vivo*

**Figure 4:**
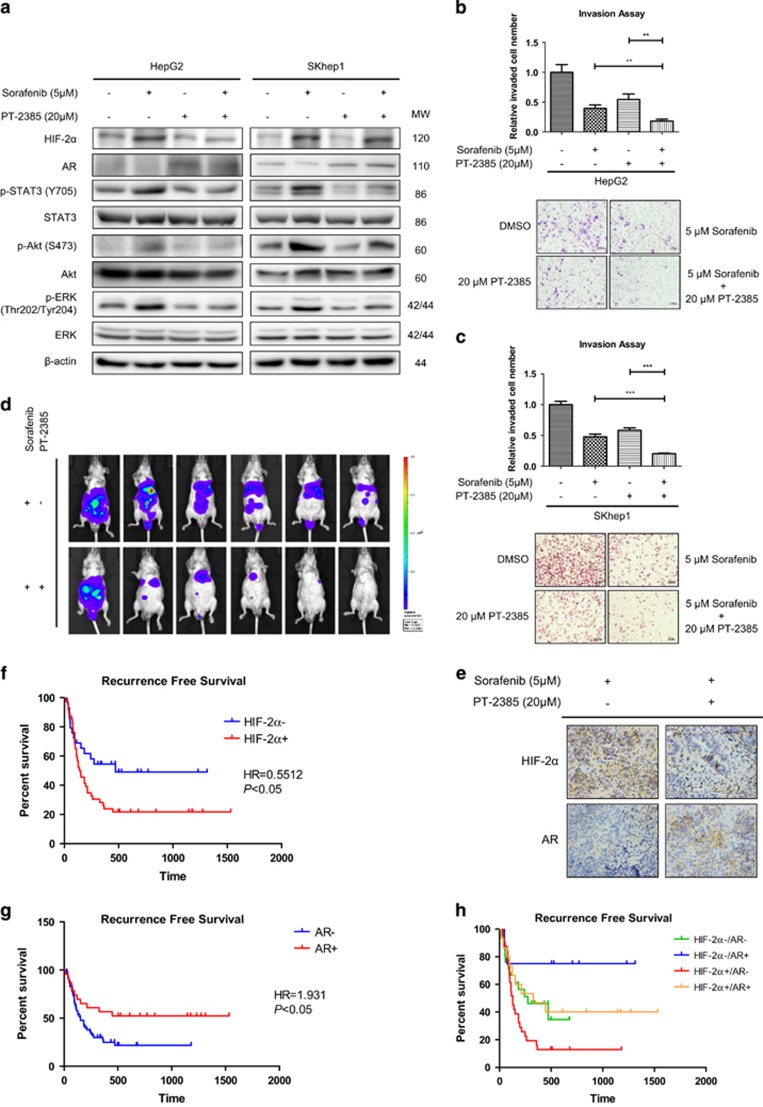
PT-2385 induces AR by suppressing HIF-2*α* and enhances sorafenib efficacy to suppress HCC invasion *in vitro* and *in vivo*. (**a**) Western blot assays showed PT-2385 could suppress HIF-2*α* level and partly reverse the decrease of AR and the increase of downstream signals (pSTAT3, pAKT and pERK) by sorafenib treatment in HepG2 and SKhep1 cells. (**b**, **c**) Chamber-transwell invasion assays suggested that PT-2385 could significantly enhance sorafenib efficacy to suppress HCC invasion in HepG2 and SKhep1 cells. Lower panel, representative images of the chamber-transwell invasion assays; upper panel, quantification of the invaded cells. The invaded cells were counted in 10 randomly chosen microscopic fields (× 100) of each experiment and pooled. (**d**) *In vivo* orthotopic mice model indicated that combined therapy of sorafenib with PT-2385 significantly increase the efficacy compared with sorafenib alone. (**e**) IHC suggested that PT-2385 could suppress HIF-2*α* and increasing AR in *in vivo* orthotopic tumors. (**f–h**) Recurrence-free survival curve of HCC patients who received surgery (*N*=75) indicated that patients with HCC (HIF-2*α*−) (defined by IHC staining) had significant higher recurrence-free survival (HR=0.5512, (**f**) than patients with HCC (HIF-2*α*+); the patients with HCC (AR+) (defined by IHC staining) had significant better recurrence-free survival (HR=1.931, (**g**) than patients with HCC (AR−); significantly better recurrence-free survival in HIF-2*α*-/AR+ group than that in HIF-2*α*+/AR− group (*P*=0.0086, HR=0.3276, (**h**). *P*<0.05 was considered statistically significant. **P < 0.01 and ***P < 0.001

As was shown above, HIF-2*α* could be induced by sorafenib treatment and could decreased AR expression, we hypothesized that PT-2385, a specific HIF-2*α* inhibitor, could induce AR by suppressing HIF-2*α* and therefore enhance sorafenib efficacy. Western blot assays showed PT-2385 could suppress HIF-2*α* level and partly reverse the decrease of AR and the increase of downstream signals (pSTAT3, pAKT and pERK) by sorafenib treatment (Figure 4a and Supplementary Figure S1D). The effects of PT-2385 on the sorafenib-targeted molecules (VEGFR2, PDGFR*β*, B-Raf and Raf1) were investigated in HepG2, SKhep1 and Huh7 cells (Supplementary Figure S2A), and results showed that PT-2385 could significantly suppress B-Raf and Raf1. The effects of PT-2385 on VEGFR2 were not consistent among three cell lines, however, it was found that PT-2385 could increase the expression of PDGFR*β*. Silencing HIF-2*α*/VEGF and HIF-2*α*/PDGF signal pathways could possibly increase the expression of their receptors (VEGFR2 and PDGFR*β*), which was considered as the feedback regulation. Invasion assays suggested that PT-2385 could significantly enhance sorafenib efficacy to suppress HCC invasion in HepG2, SKhep1 and Huh7 cells (Figures 4b and c and Supplementary Figure S1E). Moreover, *in vivo* orthotopic mice model also indicated that combined therapy of sorafenib with PT-2385 significantly increase the efficacy compared with sorafenib alone by suppressing HIF-2*α* and increasing AR (Figures 4d and e). Furthermore, immunohistochemistry (IHC) of clinical samples from sorafenib treated HCC patients indicated better recurrence-free survival in HIF-2*α* negative and AR positive groups (Figures 4f and g). Further RFS analysis based on HIF-2*α*+/AR+, HIF-2*α*+/AR−, HIF-2*α*-/AR+, and HIF-2*α*-/AR− groups suggested significantly better recurrence-free survival in HIF-2*α*-/AR+ group than that in HIF-2*α*+/AR− group (*P*=0.0086, HR=0.3276, Figure 4h). Typical IHC stainings of HIF-2*α* and AR were in Supplementary Figure 2B.

**Figure 5:**
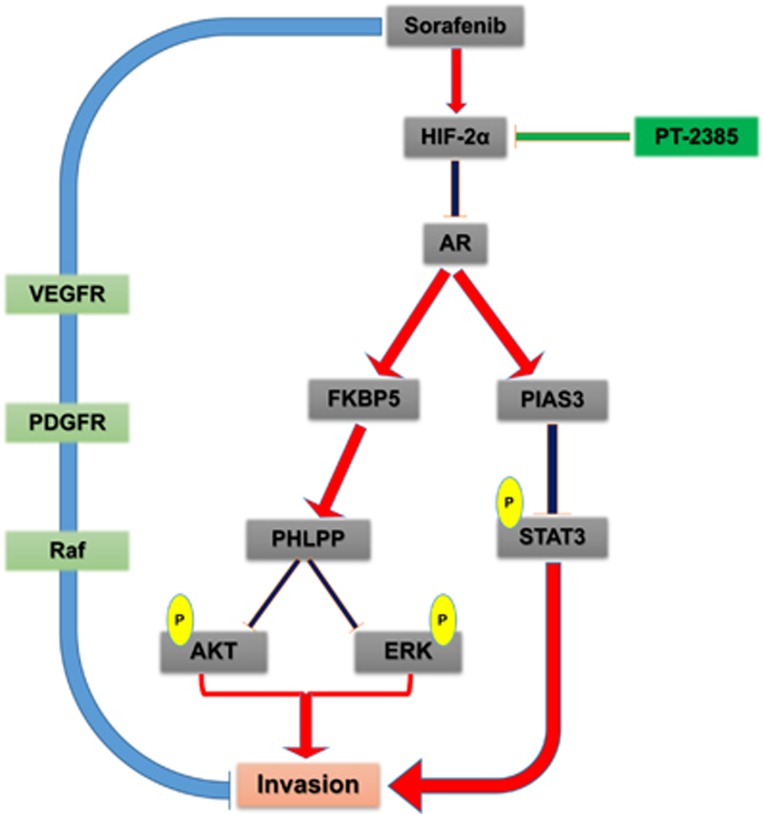
Schematic depiction. The schematic depiction showing the sorafenib-induced or hypoxia-induced HIF-2*α* could transcriptionally suppress AR and consequently activated its downstream pSTAT3/pAKT/pERK signals. Importantly, a specific and potent HIF-2*α* inhibitor, PT-2385, could significantly enhance sorafenib efficacy by suppressing HIF-2*α*, increasing AR and suppressing downstream pSTAT3/pAKT/pERK pathways

These findings strongly suggested that PT-2385 could overcome the unwanted rebounds of HCC sorafenib treatment by suppressing HIF-2*α* and consequently increasing AR (Figure 5). The sorafenib-PT-2385 combination therapy might shed light for late-stage HCC patients.

## Discussion

HCC is the most common primary liver malignancy, accounting for estimated 70–85% of the total liver cancer burden worldwide.^27, 28^ HCC is usually asymptomatic at early stages, whereas most patients are diagnosed too advanced for surgical intervention, when therapies are less effective.^29^ Indeed, most patients die within 1 year of diagnosis largely owing to recurrence and metastases, even patients who have received liver resection often have a high frequency of recurrence and metastases, propelling a rapid development of life-prolonging therapies.^30^ In recent years, molecularly targeted therapy with sorafenib yield promising results, representing a unique breakthrough on fighting late-stage HCC. The sorafenib HCC Assessment Randomized Protocol (SHARP) trial^4^ and the Phase III sorafenib Asia-Pacific (AP) trial^5^ disclosed 2.3–3 months prolongation on median overall survival time by sorafenib administration. However, low response rate, transient and limited efficacy, primary and acquired resistance and negative side-effects gradually came into focus since sorafenib was widely administered,^4, 6, 7, 8, 9, 10, 31, 32, 33^ suggesting the need for better efficacious combination therapy.

As a result of the defective vascularization and intensive metabolism, hypoxia remains a prominent feature in solid tumors including HCC, associating with recurrence, metastases and chemo- or radio-resistance.^34^ In particular, the anti-angiogenic activity of sorafenib could lead to tumor starvation and subsequent hypoxia with the tumor, provoking poor response to sorafenib and even drug resistance. Indeed, a previous study found the administration of sorafenib could resulted vasculature decrease and subsequently led to elevated tumor hypoxia within short-term treatment.^35^ Importantly, as reported, accelerated tumor growth accompanied with increased tumor hypoxia in subcutaneous and orthotopic tumor models suggested that hypoxia was able to induce sorafenib resistance by decreasing the growth inhibition and apoptosis mediated by sorafenib.^13^ Therefore, therapy induced hypoxia-related signals are promising targets for combined therapies improving sorafenib efficacy.

Cells adapt to low oxygen through an orchestrated transcriptional response regulated by HIFs, which regulate numerous signaling events involved in HCC metastasis by binding specific DNA sequences known as HREs in target genes.^36, 37, 38^ Both HIF-1*α* and HIF-2*α* were reported to control HCC progression, metastasis and chemo-sensitivity.^39, 40^ Studies found that the anti-angiogenic activity by sorafenib in HCC inhibited HIF-1*α* synthesis, and subsequently upregulated HIF-2*α* through a reciprocal regulatory mechanism, contributing to the sorafenib resistance.^26^ Indeed, earlier study had already disclosed that the mechanism of a switch from HIF-1*α*- to HIF-2*α*-dependent ways, providing more aggressive tumor growth under hypoxia condition.^39^ These findings strongly indicated a preferential therapeutic target of HIF-2*α* for HCC management.

Owing to prominent male predominance in HCC morbidity, AR was believed to be a promoter for hepatocarcinogenesis.^15^ However, early clinical trials came to inconsistent conclusions when treating HCC patients with diverse anti-androgen therapies.^41, 42, 43^ The dual roles of AR in HCC were recently revealed that AR promoted the initiation and development at early stages, whereas functioned as a metastasis suppressor at late stages, which was supported by *in vitro* cell line studies, *in vivo* mouse model studies, bioinformatics analyses and human clinical evidences, indicating that AR should be stage-dependently targeted in the treatment of HCC.^16, 17^ Unfortunately, we found that AR was downregulated upon sorafenib treatment, which in turn weakened the effect of sorafenib on suppressing invasion. In addition, exogenously introducing AR could enhance the therapeutic effect of sorafenib to suppress HCC invasion via AR-pSTAT3/pAKT/pERK pathway. Particularly, the underlying mechanisms of AR reduction by sorafenib should be vital for better efficacy.

In the present study, we found that the sorafenib-induced or hypoxia-induced HIF-2*α* could bind to an HRE within 500 bp region of AR promoter and thus transcriptionally suppress AR. Importantly, we found a specific and potent HIF-2*α* inhibitor, PT-2385, could significantly enhance sorafenib efficacy by suppressing HIF-2*α*, increasing AR and suppressing downstream pSTAT3/pAKT/pERK pathways. It is promising that PT-2385 could alleviate the undesirable side-effects of sorafenib treatment by sorafenib-PT-2385 combination therapy, which may shed light for late-stage HCC patients.

## Materials and methods

### Materials

Sorafenib was purchased from Selleck Chemicals (Houston, TX, USA). PT-2385 was purchased from MedChemexpress (Monmouth Junction, NJ, USA). Cobalt chloride was purchased from Aladdin (Ontario, CA, USA). Oxyrase was purchased from Oxyrase, Inc. (West Mansfield, OK, USA).

### *In vitro* cell culture/maintenance

The human HCC cell line HepG2 (RRID: CVCL_0027) and SKhep1 (RRID: CVCL_0525) were purchased from the American Type Culture Collection (ATCC, Manassas, VA, USA). All the cell lines were cultured in Dulbecco's Modified Eagle's Media (Invitrogen, Grand Island, NY, USA) supplemented with 10% fetal bovine serum (FBS) (v/v), penicillin (25 units/ml), streptomycin (25 g/ml), 1% l-glutamine, and 10% FBS. Both cell lines were cultured in a 5% (v/v) CO_2_ humidified incubator at 37 °C. To induce hypoxia, cells were incubated in an atmosphere of 1% O2, 5% CO2, and 94% N_2_ at 37 °C.

### Tissue samples

For mouse study, we collected all the livers of the mice and carefully examine the HCC nodules in them by H&E staining, and at least one nodule per liver were included although some of them are quite small for treatment group. For clinical samples, tissue microarray (Super Biotek, Shanghai, China) was applied with totally 80 HCC samples from the patients treated with sorafenib. Among the samples, three of them lack the recurrence information and two of them were stain without cell nucleus. Thus, totally 75 samples were included in this study.

### Invasion assay

The invasion capability of HCC cells was determined by the chamber-transwell invasion assay. The upper chambers of 8 *μ*m-pore-size polycarbonate membrane filters (Corning, Inc., Corning, NY, USA) were pre-coated with diluted growth factor-reduced matrigel (1:14 serum-free DMEM) (BD Biosciences, San Jose, CA, USA). Before invasion assays, HCC cells were plated in six-well plates at 5 × 10^5^ cells/well and treated as designated for 48 h. Then the cells were harvested by trypsinization and 1 × 10^5^ HepG2 cells or 5 × 10^4^ SKhep1 cells in serum-free DMEM were plated into the upper chambers and 700 *μ*l 10% FBS media was placed in the lower chambers for incubation at 37 °C in 5% (v/v) CO_2_ incubator for 24 h. After incubation, the cells in the upper chamber were removed and membranes scrapped and the cells invaded into the lower part of the membranes were stained with 0.1% (w/v) crystal violet. The invaded cells were counted in ten randomly chosen microscopic fields (× 100) in each experiment and averaged for quantification.

### 3D invasion assay

In brief, 5 × 10^4^ cells in 3 ml media containing 2.5% Matrigel and 30 ng EGF were plated into the collagen/Matrigel mixture-coated plate. After treating for 48 h, the media were replenished and every 3 days afterwards for 10 days. The cells with protrusion were regarded as invaded cells and 10 random different fields under × 200 magnification were counted.

### Western blot analysis

Cells were lysed in lysis buffer and proteins (30 *μ*g) were separated on 10–12% SDS/PAGE gel and then transferred onto PVDF membranes (Millipore, Billerica, MA, USA). After blocking membranes, they were incubated with appropriate dilutions of specific primary antibodies against *β*-actin (from Sigma-Aldrich, St. Louis, MO, USA), Phospho-STAT3 (Tyr705), pAKT(S473), AKT, Phospho-p44/42 MAPK (Erk1/2) (Thr202/Tyr204), p44/42 MAPK (Erk1/2) (from Cell Signaling, Danvers, MA, USA), FKBP5, PHLPP (from Bethyl, Montgomery, TX, USA), AR, STAT3 (from Santa Cruz, Dallas, TX, USA), and HIF-2*α* (from Abcam, Cambridge, MA, USA). The blots were incubated with horseradish peroxidase (HRP)-conjugated secondary antibodies and visualized using the ECL system (Thermo Fisher Scientific, Rochester, NY, USA).

### RNA extraction, miRNA extraction, reverse transcription and quantitative real-time PCR analysis

For RNA extraction, total RNAs were isolated using Trizol reagent (Invitrogen). A total of 1 *μ*g RNA was subjected to reverse transcription using Superscript III transcriptase (Invitrogen). Quantitative real-time PCR (qRT-PCR) was conducted using a Bio-Rad CFX96 system with SYBR green to determine the mRNA expression level of a gene of interest. Expression levels were normalized to the expression of GAPDH RNA.

### Chromatin immunoprecipitation assay

Cell lysates were precleared sequentially with normal rabbit IgG (sc-2027, Santa Cruz Biotechnology) and protein A-agarose. Anti-HIF-2*α* antibody (Abcam) was added to the cell lysates and incubated at 4 °C overnight. For the negative control, IgG was used. Specific primer sets designed to amplify a target sequence within the human AR promoter (ranges 500 bp upstream of the AR 5′-UTR) containing HRE are forward: 5′-GCA GGA GCT ATT CAG GAA GCA-3′, reverse: 3′-GCA AAT GCA ACA GTT TGC GAG-5′. PCR products were identified by agarose gel electrophoresis.

### Plasmid construction and luciferase assay

The 500 bp promoter of AR was chemically synthesized and ligased into GV238 backbone. For the HRE mutation, we designed the primers as:

F1 5′-TTTCTCTATCGATAGGTACCCAGCAAGTATCTGCTGGCTTGG-3′;

R1 5′-CTTAGATCGCAGATCTCGAGGAGGGGGCGCTGGGAGGTGGAG-3′

F2 5′-GCCCTCGCCAAGCTTGCGCCAGCACTTGTTTCTCC-3′;

R2 5′-TGGCGCAAGCTTGGCGAGGGCAGGAGAGGCTAG-3′

Overlap PCR was applied to generate the mutation. For the luciferase assay, cells were plated in 24-well plates and the cDNA transfected using Lipofectamine 3000 (Invitrogen) according to the manufacturer's instruction. pRL-TK was used as internal control. Luciferase activity was measured by Dual-Luciferase Assay (Promega, Madison, WI, USA) according to the manufacturer's manual. The detailed sequences of wild type and mutant 500 bp AR promoter could be found in Supplementary Table 1.

### Lentiviral-based gene delivery

The pWPI-HIF-2*α* plasmid was cloned following Gibson Assembly protocols. The pWPI, pWPI-HIF-2*α*, the psAX2 packaging plasmid, and pMD2G envelope plasmid, were transfected into 293 T cells using the standard calcium phosphate transfection method for 48 h to get the lentivirus soups. The lentivirus soups were collected and concentrated by density gradient centrifugation, then frozen at −80 °C for later use.

### *In vivo* studies

Sixteen 4–6 weeks old athymic nude BALB/c (nu/nu) male mice were purchased from Shanghai Laboratory Animal Center (SLAC). Intrahepatic injections of 1 × 10^6^ SKhep1-luc cells/100 *μ*l serum-free DMEM and matrigel (1:1) were performed on each nude mouse. Cells were first prepared as stable luciferase clones by stable infection of Luciferase lentivirus and were selected with G418 and expanded in culture. One month later, the mice were divided into experimental groups according to tumor size following *in vivo* imaging (IVIS Spectrum, Caliper Life Sciences, Hopkinton, MA, USA) after injecting 150 mg/kg Luciferin in tail vein, to start the treatment with a similar mean size in each group: (1) sorafenib treatment alone; (2) sorafenib combined with PT-2385 treatment. The mice were treated with/without sorafenib (30 mg/kg/mice; every another day, I.P.) and with/without PT-2385 (60 mg/kg/mice; every another day, I.P.) for another month. All control mice receive an equal volume of carrier solution by I.P. Tumor development/response was then monitored by IVIS once a week. The mice were killed 4 weeks after treatment and tumors and any metastases were removed for studies. All animal experiments were performed humanely in compliance with guidelines reviewed by the Animal Ethics Committee of the Biological Resource Centre of the Agency for Science, Technology and Research at the Sir Run-Run Shaw Hospital.

### H&E and IHC staining

Tissues were fixed in 10% (v/v) formaldehyde in PBS, embedded in paraffin, and cut into 5 *μ*m sections and used for H&E staining and IHC staining with specific primary antibodies against AR (Santa Cruz), and HIF-2*α* (Abcam). To enhance antigen exposure, the slides were treated with 1 × EDTA at 98 °C for 10 min for antigen retrieval. The slides were incubated with endogenous peroxidase blocking solution, and then were incubated with the primary antibody at 4 °C overnight. After rinsing with Tris-buffered saline, the slides were incubated for 45 min with biotin-conjugated secondary antibody, washed, and then incubated with enzyme conjugate HRP-streptavidin. Freshly prepared DAB (Zymed, South San Francisco, CA, USA) was used as substrate to detect HRP. Finally, slides were counter-stained with hematoxylin and mounted with aqueous mounting media. Positive cells were calculated as the number of immunopositive cells × 100% divided by total number of cells/field in 10 random fields at × 400 magnification. The slides were reviewed and scored by an experienced pathologist without the knowledge of patient outcome. The expression of AR and HIF-2*α* was assessed semiquantitatively as follows: negative (−) <5%, 5–25% (+, weak positive), 25–50% (++, positive) and >50% (+++, strong positive). Negative and weakly positive expression were defined as low expression, whereas positive and strong positive expression were defined as high expression.

### Statistical analysis

Data are expressed as mean±S.E.M. from at least three independent experiments. Statistical analyses involved Student’s *t*-test with GraphPad Prism 5 (GraphPad Software, Inc., La Jolla, CA, USA). *P*<0.05 was considered statistically significant.
