# Supplementary material for: Increasing AR by HIF-2α inhibitor (PT-2385) overcomes the side-effects of sorafenib by suppressing hepatocellular carcinoma invasion via alteration of pSTAT3, pAKT and pERK signals
Source: Cell Death Dis. 2017 Oct 12;8(10):e3095–. doi: 10.1038/cddis.2017.411 (PMC5680567; doi:10.1038/cddis.2017.411)
Supplement: Supplementary Figures [file cddis2017411x1.ppt]

## Slide 1
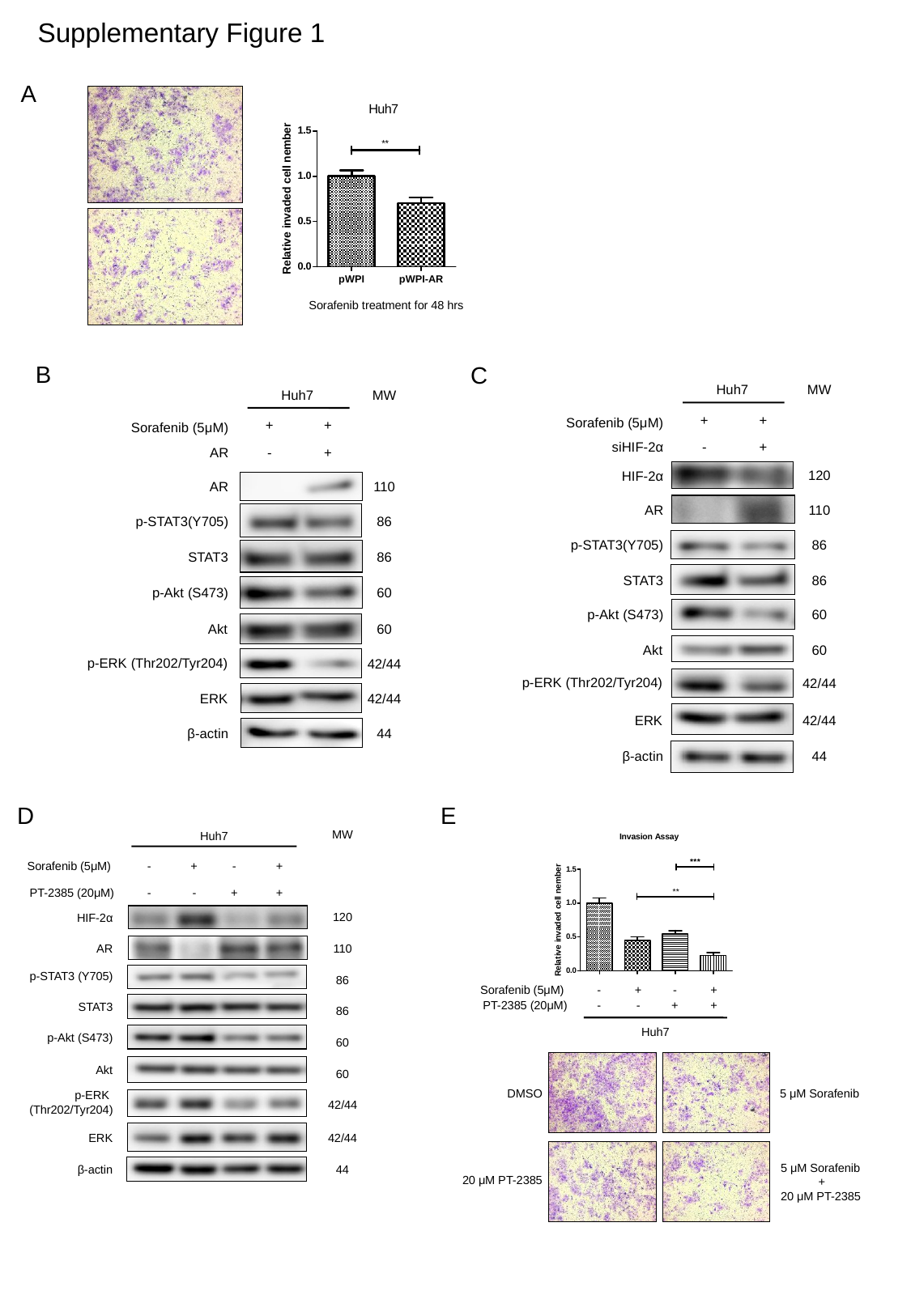

Supplementary Figure 1
A
Sorafenib treatment for 48 hrs
B
C
MW
Huh7
+
+
Sorafenib (5μM)
-
+
siHIF-2α
120
HIF-2α
AR
110
p-STAT3(Y705)
86
STAT3
86
p-Akt (S473)
60
Akt
60
p-ERK (Thr202/Tyr204)
42/44
ERK
42/44
β-actin
44
MW
Huh7
+
+
Sorafenib (5μM)
-
+
AR
AR
110
p-STAT3(Y705)
86
STAT3
86
p-Akt (S473)
60
Akt
60
p-ERK (Thr202/Tyr204)
42/44
ERK
42/44
β-actin
44
D
MW
120
110
86
86
60
60
42/44
42/44
44
Huh7
-
+
-
+
Sorafenib (5μM)
PT-2385 (20μM)
-
-
+
+
HIF-2α
AR
p-STAT3 (Y705)
STAT3
p-Akt (S473)
Akt
p-ERK
(Thr202/Tyr204)
ERK
β-actin
E
-
+
-
+
Sorafenib (5μM)
PT-2385 (20μM)
-
-
+
+
Huh7
DMSO
5 μM Sorafenib
5 μM Sorafenib
 +
20 μM PT-2385
20 μM PT-2385

## Slide 2
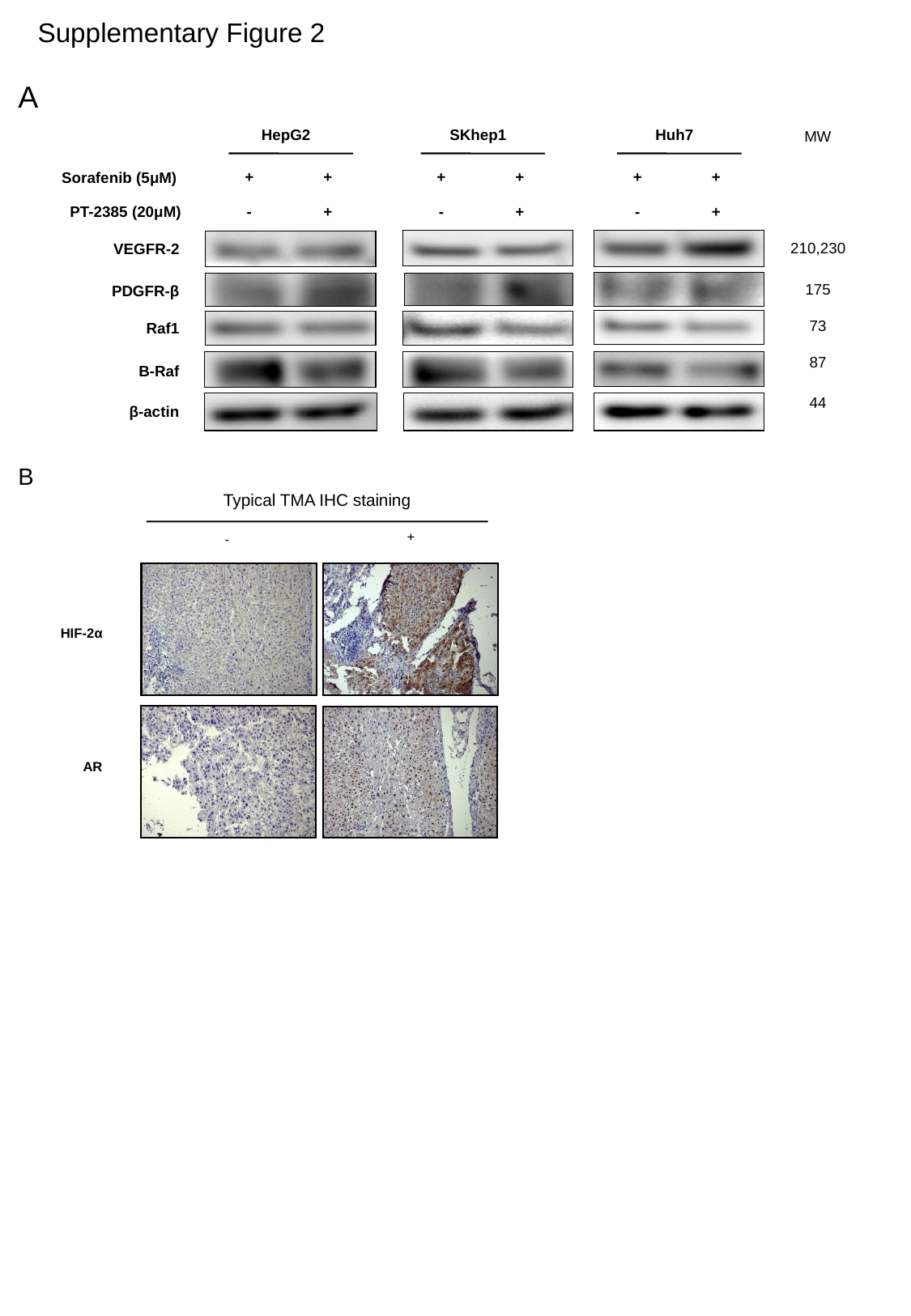

Supplementary Figure 2
A
HepG2
SKhep1
Huh7
MW
210,230
175
73
87
44
+
+
+
+
+
+
Sorafenib (5μM)
PT-2385 (20μM)
-
+
-
+
-
+
VEGFR-2
PDGFR-β
Raf1
B-Raf
β-actin
B
 Typical TMA IHC staining
+
-
HIF-2α
AR
