# Supplementary material for: Increasing AR by HIF-2α inhibitor (PT-2385) overcomes the side-effects of sorafenib by suppressing hepatocellular carcinoma invasion via alteration of pSTAT3, pAKT and pERK signals
Source: Cell Death Dis. 2017 Oct 12;8(10):e3095–. doi: 10.1038/cddis.2017.411 (PMC5680567; doi:10.1038/cddis.2017.411)
Supplement: Supplementary Figure Legends [file cddis2017411x2.docx]

Legends to Supplementary Figures

**Supplementary Figure 1:**

**(A)**, chamber-transwell invasion assays showed that over-expression of AR decreased the cell invasion in Huh7 cells under 48h Sorafenib (5μM) treatment. Left panel, representative images of the chamber-transwell invasion assays; right panel, quantification of the invaded cells. The invaded cells were counted in 10 randomly chosen microscopic fields (100×) of each experiment and pooled. **(B-C)**, western blot assays were used to test downstream altered molecules upon over-expressing AR and siHIF-2α in Huh7 cells under 48h Sorafenib treatment. **(D)**, western blot assays showed PT-2385 could suppress HIF-2α level and partly reverse the decrease of AR and the increase of downstream signals (p-STAT3, p-AKT and p-ERK) by Sorafenib treatment in Huh7 cells. **(E)**, chamber-transwell invasion assays suggested that PT-2385 could significantly enhance Sorafenib efficacy to suppress HCC invasion in Huh7 cells. Lower panel, representative images of the chamber-transwell invasion assays; upper panel, quantification of the invaded cells. The invaded cells were counted in 10 randomly chosen microscopic fields (100×) of each experiment and pooled. *p*<0.05 was considered statistically significant. * *p*<0.05, ** *p*<0.01 and *** *p*<0.001.

**Supplementary Figure 2:**

**(A)**, the effect of PT-2385 on the sorafenib-targeted molecules (VEGFR2, PDGFRβ, B-Raf and Raf1) was investigated in HepG2, SKhep1 and Huh7 cells by western blot. **(B)**, typical IHC stainings of HIF-2α and AR were shown.
