# Supplementary material for: Increasing AR by HIF-2α inhibitor (PT-2385) overcomes the side-effects of sorafenib by suppressing hepatocellular carcinoma invasion via alteration of pSTAT3, pAKT and pERK signals
Source: Cell Death Dis. 2017 Oct 12;8(10):e3095–. doi: 10.1038/cddis.2017.411 (PMC5680567; doi:10.1038/cddis.2017.411)
Supplement: Supplementary Table 1 [file cddis2017411x3.docx]

Supplementary Table 1.

Sequence of wild type 500bp AR promoter:

CAGCAAGTATCTGCTGGCTTGGTCATGGCTTGCTCCTCAGTTTGTAGGAGACTCTCCCACTCTCCCATCTGCGCGCTCTTATCAGTCCTGAAAAGAACCCCTGGCAGCCAGGAGCAGGTATTCCTATCGTCCTTTTCCTCCCTCCCTCGCCTCCACCCTGTTGGTTTTTTAGATTGGGCTTTGGAACCAAATTTGGTGAGTGCTGGCCTCCAGGAAATCTGGAGCCCTGGCGCCTAAACCTTGGTTTAGGAAAGCAGGAGCTATTCAGGAAGCAGGGGTCCTCCAGGGCTAGAGCTAGCCTCTCCTGCCCTCGCC**CACGC**TGCGCCAGCACTTGTTTCTCCAAAGCCACTAGGCAGGCGTTAGCGCGCGGTGAGGGGAGGGGAGAAAAGGAAAGGGGAGGGGAGGGAAAAGGAGGTGGGAAGGCAAGGAGGCCGGCCCGGTGGGGGCGGGACCCGACTCGCAAACTGTTGCATTTGCTCTCCACCTCCCAGCGCCCCCTC

HRE: CACGC

Sequence of mutant 500bp AR promoter:

CAGCAAGTATCTGCTGGCTTGGTCATGGCTTGCTCCTCAGTTTGTAGGAGACTCTCCCACTCTCCCATCTGCGCGCTCTTATCAGTCCTGAAAAGAACCCCTGGCAGCCAGGAGCAGGTATTCCTATCGTCCTTTTCCTCCCTCCCTCGCCTCCACCCTGTTGGTTTTTTAGATTGGGCTTTGGAACCAAATTTGGTGAGTGCTGGCCTCCAGGAAATCTGGAGCCCTGGCGCCTAAACCTTGGTTTAGGAAAGCAGGAGCTATTCAGGAAGCAGGGGTCCTCCAGGGCTAGAGCTAGCCTCTCCTGCCCTCGCC**AAGCT**TGCGCCAGCACTTGTTTCTCCAAAGCCACTAGGCAGGCGTTAGCGCGCGGTGAGGGGAGGGGAGAAAAGGAAAGGGGAGGGGAGGGAAAAGGAGGTGGGAAGGCAAGGAGGCCGGCCCGGTGGGGGCGGGACCCGACTCGCAAACTGTTGCATTTGCTCTCCACCTCCCAGCGCCCCCTC

Mutant site: AAGCT
